# Screening for *FMR1* CGG Repeat Expansion in Thai Patients with Autism Spectrum Disorder

**DOI:** 10.1155/2021/4359308

**Published:** 2021-12-08

**Authors:** Areerat Hnoonual, Charunee Jankittunpaiboon, Pornprot Limprasert

**Affiliations:** ^1^Department of Pathology, Faculty of Medicine, Prince of Songkla University, Songkhla 90110, Thailand; ^2^Office of Scientific Instrument and Testing, Prince of Songkla University, Songkhla 90110, Thailand; ^3^Faculty of Medicine, Siam University, Bangkok 10160, Thailand

## Abstract

Autism spectrum disorder (ASD) is a complex disorder with a heterogeneous etiology. Fragile X syndrome (FXS) is recognized as the most common single gene mutation associated with ASD. FXS patients show some autistic behaviors and may be difficult to distinguish at a young age from autistic children. However, there have been no published reports on the prevalence of FXS in ASD patients in Thailand. In this study, we present a pilot study to analyze the CGG repeat sizes of the *FMR1* gene in Thai autistic patients. We screened 202 unrelated Thai patients (168 males and 34 females) with nonsyndromic ASD and 212 normal controls using standard FXS molecular diagnosis techniques. The distributions of *FMR1* CGG repeat sizes in the ASD and normal control groups were similar, with the two most common alleles having 29 and 30 CGG repeats, followed by an allele with 36 CGG repeats. No *FMR1* full mutations or premutations were found in either ASD individuals or the normal controls. Interestingly, three ASD male patients with high normal CGG and intermediate CGG repeats (44, 46, and 53 CGG repeats) were identified, indicating that the prevalence of *FMR1* intermediate alleles in Thai ASD patients was approximately 1% while these alleles were absent in the normal male controls. Our study indicates that CGG repeat expansions of the *FMR1* gene may not be a common genetic cause of nonsyndromic ASD in Thai patients. However, further studies for mutations other than the CGG expansion in the *FMR1* gene are required to get a better information on FXS prevalence in Thai ASD patients.

## 1. Introduction

Autism spectrum disorder (ASD) is a complex neurodevelopmental disorder characterized by deficits in social interactions and social communications, as well as restricted interests and stereotyped and repetitive behaviors. In recent years, an increase in the apparent prevalence of autism has been reported worldwide. The prevalence of ASD was estimated to be 1 in 54 children in a recent study, with a 4 : 1 ratio of affected males to females [1]. The diagnosis of ASD is primarily based on medical assessment, behavioral evaluations, and the application of the autism criteria enumerated in the Diagnostic and Statistical Manual of Mental Disorders (DSM-5). The causes of ASD remain largely unknown, but an earlier study in twins reported a consistently high genetic contribution to ASD [2]. It is generally thought that ASD is likely explained by a multifactorial etiology which includes various inherited factors, genetic mutations, and environmental factors. About 10-20% of ASD cases have been identified as having a genetic disorder caused by chromosomal aberrations and single gene mutations, including fragile X syndrome, tuberous sclerosis, and Rett syndrome. More than 100 ASD-risk genes carrying mutations have been identified in ASD [3–8]; therefore, genetic testing is indicated in the medical workup for individuals with ASD, which may include G-banded karyotyping, fragile X testing, chromosomal microarray, and/or whole exome sequencing [5, 9].

Fragile X syndrome (FXS) is the most frequent monogenic cause of intellectual disability (ID) and ASD. FXS is caused by expansion of CGG repeats in the 5′ untranslated region of the fragile X mental retardation 1 gene (*FMR1*), which is located on chromosome Xq27.3. It has been established that the normal CGG repeat number is below 45 and alleles in this repeat range are transmitted stably from generation to generation. The AGG intersperse the CGG repeat every 9 or 10 CGG repeats, reducing repeat instability [11, 12]. CGG repeats between 55 and 200 are considered FMR1 premutation alleles, which are associated with maternal expansions of the number of CGGs in the next generation. More than 200 repeats result in a full mutation which gives rise to FXS. The majority of males who carry the full mutation FXS have mild to moderate ID, while about half of females with the full mutation have only normal or borderline intellectual function because of cellular mosaicism resulting from X-chromosome inactivation. As with most aspects of the FXS phenotype, behavioral phenotypes in FXS are quite variable and include attention deficits, hyperactivity, hyperarousal, aggression and self-injury, social anxiety, and autism [13, 14]. Some studies have reported that some behavioral symptoms of patients with FXS are similar to autistic patients and it is sometimes difficult to distinguish them from each other, especially in young children [15–18]. FXS is the most commonly known inherited single-gene cause of ASD, accounting for approximately 1-6% of all autistic cases [5, 9, 17, 19]. Over the past decade, many studies have evaluated the FXS-ASD link. In individuals with the full FXS mutations, up to 60% of males meet the diagnostic criteria for ASD and approximately 30-50% of males with FXS meet the full DSM-IV criteria for autism [17, 20–25]. Additionally, more than 90% of males with FXS display some autistic characteristics [14, 16, 17]. The percentage of autism is lower (3-20%) in females with FXS [17, 21, 26, 27]. In recent years, changes in the diagnostic criteria for ASD from DSM-IV to DSM-5 have resulted in slightly changed incidence rates. Approximately 50% of males and 30% of females with FXS who met the full criteria for an ASD diagnosis using the DSM-IV also meet the DSM-5 criteria [28]. Although autistic features show high prevalences in FXS, the fact is that sometimes a patient will have no signs of the syndrome, therefore, all children affected by ASD, especially boys, should be tested for *FMR1* mutations. Molecular screening for CGG repeat expansions of the *FMR1* gene is now recommended for all individuals diagnosed with ASD due to the high levels of comorbidity between ASD and FXS and the variable expressiveness of FXS [9, 29–32]. Studies of *FMR1* CGG repeats using DNA testing have found widely varying levels of abnormalities in the *FMR1* gene [33–52], while some studies have found no abnormal *FMR1* genes in patients with autism [53–56].

The widely varying findings of FXS frequency raise the question as to whether CGG repeat expansions of the *FMR1* gene may not be a common cause in patients with ASD. Moreover, the rate of *FMR1* full mutations identified among ASD cohorts is generally lower than that identified in ID cohorts [43, 55, 57]. In Thailand, one study of FXS frequencies in patients with ID and DD of unknown etiology found that the frequency of a full mutation was 6.8% (16/237) [58]; however, to date, there have been no studies on FXS mutations in Thai ASD patients. Therefore, the aim of this study was to analyze the CGG repeat sizes of the *FMR1* gene in Thai patients with nonsyndromic ASD.

## 2. Materials and Methods

### 2.1. Study Cohort

A total of 202 patients with ASD (168 males and 34 females) were recruited from a cohort of 203 Thai children with nonsyndromic ASD, excluding one male patient with ring chromosome 13, reported in a previous study [59]. All patients had normal karyotypes. The mean ages in the male and female patients were 4.33 and 4.19 years, respectively. These patients met the diagnostic criteria for autistic disorder or pervasive developmental disorders-not otherwise specified (PDD-NOS) according to the DSM-IV using a clinical checklist [60]. Two hundred and twelve normal male controls were selected from a previous study [61] to compare CGG repeat distributions with ASD cases.

The study protocol was approved by the three Institutional Ethics Committees (EC 48/364-006, ID 05-49-24, and No.061/2548).

### 2.2. FXS DNA Analysis

All patients were screened for FXS using standard molecular methods according to the American College of Medical Genetics and Genomics (ACMG) Standards and Guidelines for fragile X testing [10, 30]. The lengths of the CGG repeats were determined through fluorescent PCR fragment analysis. The PCR reactions were performed in 10 *μ*l mixtures comprised of 25 ng DNA, 1X PCR buffer, 1 mM MgCl_2_, 200 *μ*M dNTPs (dGTP replaced with 7-deaza dGTP), 2.2 M betaine, 0.2 *μ*M each of the primers FRAXA-PSU-F (5′-6FAM-CAGCGTTGATCACGTGACGTGGTTTCAGTG-3′) and FRAXA-PSU-R (5′-GATGGGGCCTGCCCTAGAGCCAAGTA-3′), and 0.5 units of Hot Start Taq DNA Polymerase (Immolase, Bioline). The PCR reactions were carried out beginning with an initial hot start at 95°C for 10 min, followed by 35 cycles of denaturation at 95°C for 1 min, annealing at 66°C for 1 min, extension at 72°C for 1 minute, and a final extension of 72°C for 10 min. The PCR product was then mixed with 10 *μ*l HiDi formamide and 0.2 *μ*l LIZ500, then denatured at 95°C for 2 min and 4°C for 5 min. The PCR mixture was loaded on an ABI3130 Genetic Analyzer and analyzed using GeneMapper v3.2 software. To predict instability or expansion of *FMR1* CGG repeat alleles, AGG interruptions were determined using *FMR1* triplet repeat-primed PCR (TP-PCR) [62] in individuals carrying intermediate or premutation alleles and their mothers. The TP-PCR assay was used not only to identify AGG interruptions, but also resolves the difficulty of detecting mosaic males when using conventional/fluorescent PCR because up to ~10% of mosaicism can be detected by the TP-PCR method [63, 64]. Methylation-specific PCR was also performed in the study males to determine their methylation status based on the method used in a previous study [65].

### 2.3. Statistical Analysis

In the analysis, the samples were divided into 7 groups according to the identified common and uncommon *FMR1* CGG repeats: 17-28, 29, 30, 31-35, 36, 37-40, and 41-54 (high normal and intermediate alleles). Chi-square test was used to test for differences in allele distributions between the ASD and normal control groups. Fisher's exact test was used to compare the high and intermediate alleles (41-54 CGG repeats) between the ASD and control groups.

## 3. Results

**Figure 1:**
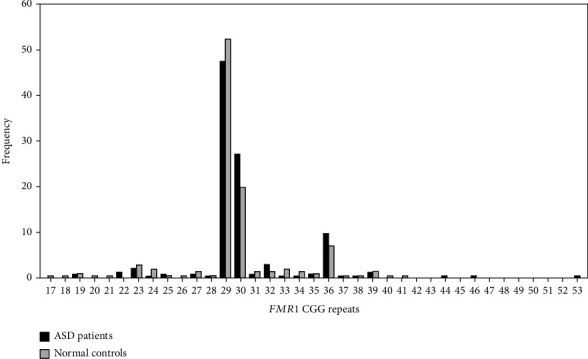
Distribution of *FMR1* CGG alleles in the study of ASD patients (*n* = 202; 168 males and 34 females) and normal Thai controls (*n* = 212 males). The most common alleles in the Thai population are 29 and 30 CGG repeats, followed by 36 repeats.

Molecular screening of FXS in the study patients with ASD revealed CGG repeats in the normal (<45 CGG repeats) or intermediate (45-54 CGG repeats) range in all ASD males (Table 1) and females (Supplementary Table 1). The allele distributions in the ASD male patients ranged from 18 to 53 CGG repeats (20 alleles) compared to 17-41 CGG repeats (24 alleles) in the male controls. Although the allele numbers in the male cases were smaller than in the male controls, the heterozygosity of alleles in the ASD male cases (0.6799) was slightly greater than that in the male controls (0.6787). Regarding the distribution of the CGG repeats, 29 and 30 repeats were the most common numbers of repeats, accounting for 50.00% and 24.40% of our ASD cases, respectively, with 36 repeats making up a further 8.93% of male ASD cases. Similarly, in the normal control group, the two most common alleles were 29 (52.36%) and 30 CGG repeats (19.81%), followed by 36 CGG repeats (7.08%) (Table 1 and Figure 1).

The allele distributions, based on the 7 groups of identified CGG repeats, between the ASD male cases and the male controls, were not statistically significantly different (chi − square = 4.787, df = 6, *P* = 0.5714, Table 1). All normal controls had *FMR1* CGG repeats in the normal range of less than or equal to 41 repeats. Interestingly, 3 male ASD patients showed high normal or intermediate CGG allele repeats of 44, 46, and 53 CGG repeats, transmitted from their mothers (Supplementary Figure 1), while these alleles were absent in the male controls. Methylation-specific PCR testing of these 3 male ASD patients with high normal or intermediate allele showed a normal pattern indicating the *FMR1* promoter unmethylated allele (Supplementary Figure 2). These 3 ASD patients and their mothers had at least one AGG interruption (Supplementary Figure 3). The presence of AGG interruptions in the CGG repeats of the *FMR1* gene reduces repeat instability during transmission from parent to child and decreases the risk of CGG expansion during maternal transmission. In our study, mothers who carried high normal or intermediate CGG alleles did not show the increase of CGG repeat expansions in their children. When we compared the high normal and intermediate CGG repeats (range: 41–54 repeats) in the male ASD cases with the male controls using Fisher's exact test, we still did not find a statistically significant difference (*P* = 0.3255). The frequency of intermediate alleles (defined as 45-54 repeats) in our ASD patients was approximately 1% (2/202). No premutations or full mutations were identified in either the ASD or normal control groups in the study.

## 4. Discussion

ASD is a group of complex neurodevelopment disorders with multiple etiologies. Among the genetic causes, mutations in the *FMR1* gene, which cause FXS, are the leading known genetic cause of autism. Diagnosis of individuals with ASD and FXS is difficult due to overlapping symptoms. Given the possibility of an ASD-FXS link, it is now recommended that all individuals with ASD should be referred for genetic evaluation and testing for FXS when the etiology of their autism is not known [5, 9]. FXS is caused by an expanded number of CGG repeats (>200 repeats) in the 5′ UTR of the *FMR1* gene leading to a deficiency or absence of FMRP, an RNA-binding protein that regulates the translation of a number of other genes that are important for synaptic development and plasticity. Many of these genes, including neuroligins, neurorexin 1, *PTEN*, *PSD95*, *MAPK1*, *JAKMIP*, *SHANK3*, and *CYFIP1*, are linked to autism when they are mutated [7, 8, 15, 66], which may explain the high comorbidity that exists between FXS and ASD. The rates of comorbid diagnosis of FXS and ASD greatly differ across the literature. The frequency of autism among males with FXS varies widely, from 18.5% in the first estimate by Brown et al. [67] and ranging from 5% to 60% in subsequent studies depending on the diagnostic criteria and methodologies used for DNA testing. However, FXS is rarely found in autism individuals who have had full clinical evaluation [17, 20–25].

The present study was aimed at screening for CGG repeat expansion in the *FMR1* gene among a group of Thai patients with nonsyndromic ASD. No premutation or full mutation alleles were found in this cohort, which is comparable to other studies done in Indonesia, Japan, Australia, and the USA [53–56, 68]. Several studies published in the last decade have reported FXS full mutations in approximately 0-6% of ASD patients [33–56] (Table 2). The rates of *FMR1* expansion among ASD patients vary widely across studies, depending on different factors including ethnic background, small sample sizes in various studies, possibility of referral bias, diagnostic criteria for autism, and method of FXS diagnosis. The discrepancies regarding the prevalence of FXS among individuals with autism may reflect the limited reliability of the cytogenetic tests used in the past compared with the more sensitive molecular tests currently used. Regarding the prevalence of FXS, there is the possibility that founder effects could result in some populations having higher prevalences of FXS. Although FXS affects all ethnic groups, the prevalence may vary between populations. A previous study reported that the incidence of FXS in countries with significant Asian populations was significantly lower than that in Western countries [69].

In our study, two ASD patients with intermediate alleles were detected, yielding an intermediate allele frequency of ~1% (2/202), which was consistent with reports in ASD patients in other populations in Italy [43] and the USA countries [47, 51, 52]. In comparing CGG repeats with a normal Thai control group, 3 ASD patients showed high normal or intermediate CGG repeat alleles, with 44, 46, and 53 CGG repeats, while these alleles were absent in the normal male controls. All three patients had delayed speech development, mild ID, and hyperactivity. The *FMR1* intermediate alleles may show some instability and may expand into the premutation range when transmitted by the mother [70, 71]. Previous studies have reported that 7.7% of parents with *FMR1* alleles in the 40-49 repeat range and 25% of parents with *FMR1* alleles in the 50-60 repeat range were more likely to pass a changed number of *FMR1* CGG repeats to their children [72]. An intermediate allele expanding to a full mutation over two generations was reported in a family where a 44 or 52 CGG maternal grandfather transmitted a full mutation to his grandson [73, 74]. The impact of alleles on intermediate CGG number is not well understood, and there are various definitions of “intermediate allele.” The American College of Medical Genetics and Genomics (ACMG) practice guidelines (2005) defined CGG repeats from 41-60 as the intermediate or gray zone [75], but the recent ACMG standards and guidelines for FXS testing changed the definition of intermediate or gray zone to 45-54 [10]. However, some population studies have used 41–54 repeats [53, 76, 77]. These discrepancies in the definition of the intermediate alleles have become more important because several studies have now reported phenotypes associated with the *FMR1* gray zone or intermediate allele. A previous study found elevated mRNA levels in intermediate alleles and premutation alleles with a lower threshold of normal CGG repeats (5-40 CGG) [78]. Based on this finding, intermediate alleles may be similar to premutation alleles which are known to be associated with some neurodevelopmental conditions and late-onset tremor ataxia (FXTAS) [79–87]. Consistent with earlier reports, we found some increase of intermediate alleles in ASD subjects compared with the controls, although the differences were nonsignificant. Some studies have reported significantly increased frequencies of intermediate alleles in individuals with autism, ID, and learning difficulties compared with normal controls [56, 88–91]; however, other studies had different findings [51, 53, 92–95]. A high frequency of intermediate alleles has also been associated with an increased risk for some behavioral phenotypes, including autistic behavior, developmental delay, and learning disabilities [84–87, 91], although such associations are uncertain as they have been based on only a few small studies. Intermediate alleles have also been identified in females with premature ovarian failure, females with Parkinsonism, and individuals with symptoms of the late-onset neurodegenerative disorder, FXTAS [79–83, 86, 87]. Despite the small sample size of the current study, our results support the hypothesis that intermediate alleles of the *FMR1* gene might be associated with autism [56, 84, 85, 91], but further studies are required with larger numbers of cases and controls from the Thai population and other different ethnic groups to confirm that intermediate alleles can be a risk factor of autism.

The CGG repeat distribution has been reported to vary widely among different populations [69]. In Thailand, this study reported the distributions of CGG alleles to be similar in both ASD and normal control groups. The most common alleles in our study group were 29 and 30 repeats, followed by 36 CGG repeats, which are the same frequencies as previously reported in the Indonesian, Chinese, Korean, and Thai populations in previous reports [58, 96–98]. The 29 and 30 CGG repeats are also the most common numbers found in Caucasian populations, but the 30 repeats are present at a higher frequency than the 29 repeats, which is different from Asian populations [69]. In contrast, a study in Japanese patients found 27, 26, and 28 to be the most common alleles [53]. In some Mexican subpopulations, 32 and 30 were the most frequent repeat numbers [99].

According to the American College of Medical Genetics and Genomics and the American Academy of Pediatrics, the first-tier genetic tests for individuals with ASD, DD, and/or ID include chromosomal microarray and fragile X testing [9, 29–32]. Moreover, several studies have suggested that the diagnostic yield of fragile X syndrome and chromosomal microarray combined with next-generation sequencing was significantly higher than that of fragile X syndrome and/or microarray alone [42, 45]. Due to the limitations of the methods commonly used for FXS diagnosis, we were unable to detect other uncommon causes of FXS including point mutations, deletions, and duplications which can also affect *FMR1* expression and FMRP level; however, during the FXS testing in our study, some available DNA samples of the patients in this cohort were also evaluated using SNP microarray and whole exome sequencing to look for point mutations, deletions, and duplications of the *FMR1* gene and other genes that may be associated with ASD in patients. During preparation of this research article, of the 202 ASD patients, SNP microarray was performed for 65 of them, and 9 patients were found to have pathogenic copy number variations (CNVs) or variants of uncertain significance (VOUS), likely pathogenic CNVs [100]. Whole exome sequencing was performed on 11 ASD patients, and two patients carrying clinically significant variants were identified [101, 102]. However, no point mutation, deletion, and duplication of the *FMR1* gene were detected in these patients. Our results and review of the literature support the recommendation that fragile X testing should be included as part of initial genetic testing in patients receiving a diagnosis of ASD. We also recommend that the combined genetic testing of *FMR1* testing, chromosomal microarray, and/or whole exome sequencing should be performed to increase the diagnostic yield of ASD.

## 5. Conclusion

This study reports for the first time the frequency of the FXS mutation in ASD patients in Thailand. Although no *FMR1* premutations or full mutations were found, the study identified high normal and intermediate alleles in three ASD patients while these alleles were absent in the normal controls. Our findings add weight to the evidence that intermediate alleles may be considered as indicating an increased risk for autism; however, further studies with larger samples of ASD cases and controls from different ethnic groups are required to further elucidate the role of intermediate alleles in the etiology of autism and other neurodevelopmental disorders. The causes of ASD could involve multiple genes and other factors, and efforts should be made to identify the causes of ASD in this group of patients. FXS may be less common in children being clinically diagnosed with ASD in the Thai population; thus, we cannot conclude from this study that the *FMR1* gene is a susceptible genetic factor in Thai autistic patients. The data from this study should provide a foundation for further investigations of FXS in Thai patients with ASD.

## Figures and Tables

**Table 1:** Comparison of CGG repeat groups between study ASD male cases and male controls.

CGG repeats	ASD males		Male controls	
	Number	Percentage	Number	Percentage
17-28	11	6.55	22	10.38
29	84	50.00	111	52.36
30	41	24.40	42	19.81
31-35	10	5.95	15	7.08
36	15	8.93	15	7.08
37-40	4	2.38	6	2.83
41-54	3^∗^	1.79	1^∗∗^	0.47
Total	168	100	212	100

^∗^44, 46, and 53 CGG repeats; ^∗∗^41 CGG repeats. Chi − square = 4.787, df = 6, *P* = 0.5714 (no statistically significant differences).

**Table 2:** Review of the literature on fragile X syndrome screening in ASD patients. Only studies that performed molecular techniques for fragile X testing are included.

Country/region	Study subjects	Instrument(s) used for ASD diagnosis	Method(s) for FXS testing	Main findings	References
*Asian countries*					
China	73 ID, DD, ADHD, ASD (28 ASD)	NA	SB	Premutation: 1.4% (1/73)	Chan and Wong [33]
China	143 ASD patients	DSM-III-R	PCR	Intermediate, premutation, full mutation: 0% (0/143)	Poon et al. [95]
China	177 ASD patients	NA	PCR	Premutation, full mutation: 4.5% (8/177)	Wang et al. [34]
Japan	109 ASD patients	DSM-IV ICD-10	PCR with CE SB	Intermediate, premutation, full mutation: 0% (0/109)	Otsuka et al. [53]
Indonesia	144 patients (i) 32 ASD (ii) 112 ID	NA	PCR with CE SB	Full mutation: 0.7% (1/144) (i) ASD: 0% (0/32) (ii) ID: 0.9% (1/112)	Winarni et al. [54]
Indonesia	65 ASD patients	DSM-IV-TR CARS	Cytogenetics PCR SB	Full mutation: 6.15% (4/65)	Winarni et al. [35]
Korea	66 ASD patients	DSM-IV CARS	SB	Premutation: 7.6% (5/66) Full mutation and mosaic mutation: 1.5% (1/66)	Kang et al. [36]
Korea	101 patients (i) 31 ASD (ii) 63 ID (iii) 7 LD	NA	Cytogenetics PCR SB	Full mutation: 1% (1/101)	Kwon et al. [37]
Sri Lanka	850 patients (i) 135 ASD (ii) 112 ADHD (iii) 603 physical and behavioral disorders	NA	PCR with CE MCA (FastFraX™ *FMR1* kit) MS-PCR SB	(i) Intermediate: 0.1% (1/850) (ii) Premutation: 0.8% (7/850) (iii) Full mutation: 1.3% (11/850) (4 ASD, 3 ADHD, 4 physical and behavioral disorders)	Chandrasekara et al. [38]
Thailand	202 ASD patients (168 males, 34 females)	DSM-IV	PCR with CE MS-PCR SB	(i) Intermediate: 1% (2/202) (ii) Premutation, full mutation: 0% (0/202)	This study

*Non-Asian countries (Australia, European, South American, North American)*					
Australia (Tasmania)	1,248 ID, ADHD, ASD	NA	PCR SB	(i) Intermediate^∗^: 3.4% (43/1,248) (ii) Premutation, full mutation: 0% (0/1,248)	Mitchell et al. [56]
Australia	16 ASD patients^∗∗^ (fragile X testing)	DSM-IV-TR	PCR SB	Full mutation: 1.2% (2/167)	Mordaunt et al. [39]
Brazil	83 ASD patients	NA	PCR with CE	(i) Intermediate: 4.8% (4/83) (ii) Premutation: 4.8% (4/83) (iii) Full mutation: 0% (0/83)	Ferreira et al. [40]
Canada	2,486 ID, DD, ASD^∗∗^	NA	NA	(i) Premutation: 0.4% (10/2,486) (ii) Full mutation and mosaic mutation: 1.2% (30/2,486)	Borch et al. [41]
France	312 ASD patients^∗∗^ (fragile X testing)	DSM ADOS CARS ADI-R	NA	Full mutation: 1.3% (4/312)	Munnich et al. [42]
Italy	2,850 patients (i) 2,750 ID, DD, hyperactivity (ii) 82 ASD (iii) 18 POI	NA	PCR with CE TP-PCR AmplideX™ FMR1 kit SB	Intermediate: 0.5% (13/2,850) Premutation: 2.9% (83/2,850) (i) ID, DD: 2.8% (77/2,750) (ii) ASD: 0% (0/82) (iii) POI: 33.3% (6/18) Full mutation: 2.9% (82/2,850) (i) ID, DD: 2.9% (81/2,750) (ii) ASD: 1.2% (1/82) (iii) POI: 0% (0/18)	Esposito et al. [43]
Israel	59 ASD patients (fragile X testing)	DSM-IV CARS	Cytogenetics SB	Full mutation: 3.4% (2/59)	Kosinovsky et al. [44]
Spain	206 ASD patients	DSM-V	PCR with CE (Asuragen AmplideX kit PCR/CE *FMR1*)	Full mutation: 1% (2/206)	Arteche-López et al. [45]
Sweden	142 ASD patients (fragile X testing)	DSM-IV ABC	NA	Full mutation: 0.7% (1/142)	Eriksson et al. [46]
USA	316 ASD patients	NA	Cytogenetics PCR SB	(i) Intermediate: 2.2% (7/316) (ii) Premutation: 0.3% (1/316) (iii) Full mutation and mosaic mutation: 1.9% (6/316)	Reddy [47]
USA	861 ASD patients^∗∗^ (fragile X testing)	DSM-IV-TR	NA	(i) Premutation: 0.2% (2/861) (ii) Full mutation and mosaic mutation: 0.2% (2/861)	Shen et al. [48]
USA	183 ASD patients^∗∗^ (fragile X testing)	DSM-IV	NA	Premutation: 1.1% (2/183) Full mutation: 0.5% (1/183)	Roesser [49]
USA	174 ASD patients^∗∗^ (fragile X testing)	ADOS ABC CARS GARS DSM-IV	NA	Full mutation: 0.6% (1/174)	McGrew et al. [50]
USA	599 patients (i) 453 autism/ASD (ii) 146 DD	ADI-R ADOS	PCR with CE TP-PCR	(i) Intermediate: 1.3% (8/599) (7 autism/ASD, 1 DD) (ii) Premutation: 0.3% (2/599) (2 DD) (iii) Full mutation: 0.7% (4/599) (2 autism, 2 DD)	Tassone et al. [51]
USA	75 ASD patients (fragile X testing)	NA	PCR with CE TP-PCR	Full mutation: 0% (0/75)	Weinstein et al. [55]
USA	299 ASD patients	DSM-V	PCR with CE (Asuragen AmplideX kit PCR/CE *FMR1*)	(i) Intermediate: 0.7% (2/299) (ii) Premutation: 1% (3/299) (iii) Full mutation and mosaic mutation: 1.3% (4/299)	Harris et al. [52]

^∗^41-60 repeats; ^∗∗^retrospective chart review. ASD: autism spectrum disorder; ABC: Autistic Behavior Checklist; ADOS: Autism Diagnostic Observation Schedule; ADI-R: Autism Diagnostic Interview-Revised; ADHD: attention deficit hyperactivity disorder; CE: capillary electrophoresis; DD: developmental delay; DSM: Diagnostic and Statistical Manual of Mental Disorders; GARS: Gilliam Autism Rating Scale; ID: intellectual disability; MCA: melting curve analysis; NA: not available; POI: Primary ovarian insufficiency; SB: southern blot analysis.

## Data Availability

All the data used to support the findings of this study are included within the article and the supplementary information file.

## Supplementary Materials

Supplementary Materials Supplementary Table 1: CGG repeat genotypes of the *FMR1* gene in ASD females. Supplementary Figure 1: fluorescent PCR fragment analysis showing 31 and 53 CGG repeats in the mother and 53 CGG repeats in the ASD son. Supplementary Figure 2: methylation-specific PCR results. Lane 1: 100 bp marker, lane 2: normal male control showing a *FMR1* promoter unmethylated band and an *XIST* promoter methylated band, lane 3: full mutation male (positive control) showing a *FMR1* promoter methylated band and an *XIST* promoter methylated band, lane 4: ASD male with 44 CGG repeats, lane 5: ASD male with 46 CGG repeats, lane 6: ASD male with 53 CGG repeats, lane: 7 normal female control, and lane 8: no template DNA. Supplementary Figure 3: electropherogram of TP-PCR for *FMR1* CGG expanded alleles and AGG interruptions. Mothers of male patients carrying high normal or intermediate CGG alleles had at least one AGG interruption: (A) full mutation female (positive control) showing serial peak; (B) female with 29 and 44 CGG repeats: 44 repeat alleles having one AGG interruption; (C) female with 29 and 46 CGG repeats: 46 repeat alleles having one AGG interruption. Superimposed close-up electropherogram shows the end of continuous stutter peaks of 46 repeats allele. (D) Female with 31 and 53 CGG repeats: 53 repeats allele having four AGG interruptions. CGG repeats are based on the fluorescent PCR results. In heterozygous females, AGG interruption patterns can only be determined in larger repeat alleles because it is difficult to correctly identify AGG patterns in lower repeats alleles.
