# Supplementary material for: Screening for FMR1 CGG Repeat Expansion in Thai Patients with Autism Spectrum Disorder
Source: Biomed Res Int. 2021 Dec 8;2021:4359308. doi: 10.1155/2021/4359308 (PMC8674057; doi:10.1155/2021/4359308)
Supplement: Supplementary Materials — Supplementary Table 1: CGG repeat genotypes of the FMR1 gene in ASD females. Supplementary Figure 1: fluorescent PCR fragment analysis showing 31 and 53 CGG repeats in the mother and 53 CGG repeats in the ASD son. Supplementary Figure 2: methylation-specific PCR results. Lane 1: 100 bp marker, lane 2: normal male control showing a FMR1 promoter unmethylated band and an XIST promoter methylated band, lane 3: full mutation male (positive control) showing a FMR1 promoter methylated band and an XIST promoter methylated band, lane 4: ASD male with 44 CGG repeats, lane 5: ASD male with 46 CGG repeats, lane 6: ASD male with 53 CGG repeats, lane: 7 normal female control, and lane 8: no template DNA. Supplementary Figure 3: electropherogram of TP-PCR for FMR1 CGG expanded alleles and AGG interruptions. Mothers of male patients carrying high normal or intermediate CGG alleles had at least one AGG interruption: (A) full mutation female (positive control) showing serial peak; (B) female with 29 and 44 CGG repeats: 44 repeat alleles having one AGG interruption; (C) female with 29 and 46 CGG repeats: 46 repeat alleles having one AGG interruption. Superimposed close-up electropherogram shows the end of continuous stutter peaks of 46 repeats allele. (D) Female with 31 and 53 CGG repeats: 53 repeats allele having four AGG interruptions. CGG repeats are based on the fluorescent PCR results. In heterozygous females, AGG interruption patterns can only be determined in larger repeat alleles because it is difficult to correctly identify AGG patterns in lower repeats alleles. [file 4359308.f1.pdf]

Supplementary Table 1. CGG repeats genotypes of the *FMR1* gene in ASD females

Genotype	Number
22,29	1
23,29	1
23,30	1
25,36	1
28,30	1
**29,29**	9
29,30	6
29,36	2
**30,30**	5
30,32	1
30,35	1
30,36	2
30,39	1
31,36	1
36,36	1
**Total**	**34**

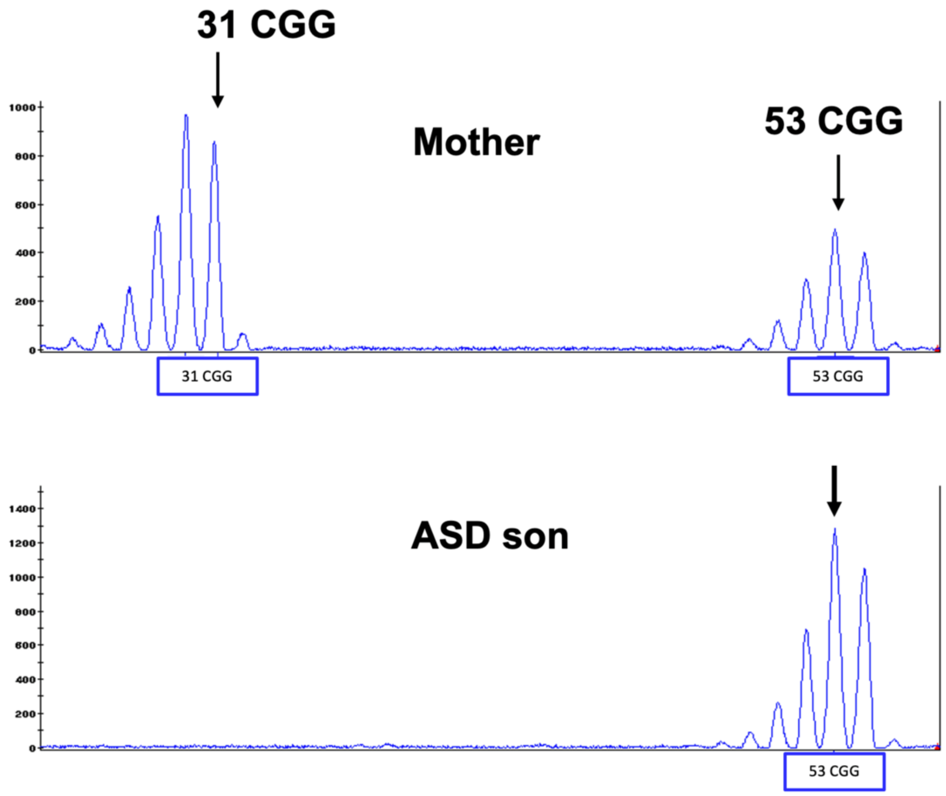

Supplementary Figure. 1. Fluorescent PCR fragment analysis showing 31 and 53 CGG repeats in the mother and 53 CGG repeats in the ASD son.

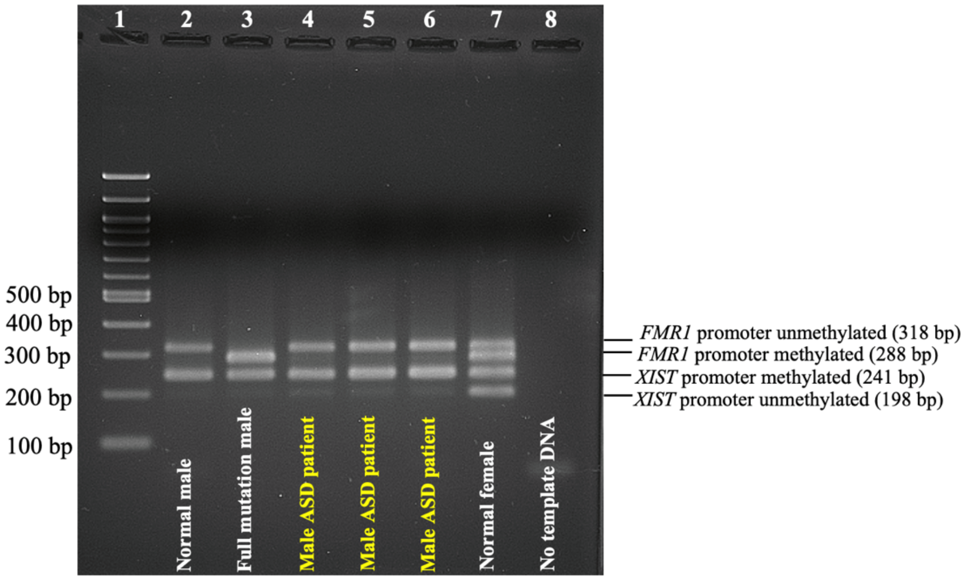

Supplementary Figure 2. Methylation-specific PCR results. Lane 1: 100 bp marker, Lane 2: normal male control showing a *FMR1* promoter unmethylated band and an *XIST* promoter methylated band, Lane 3: full mutation male (positive control) showing a *FMR1* promoter methylated band and an *XIST* promoter methylated band, Lane 4: ASD male with 44 CGG repeats, Lane 5: ASD male with 46 CGG repeats, Lane 6: ASD male with 53 CGG repeats, Lane: 7 normal female control, Lane 8: no template DNA.

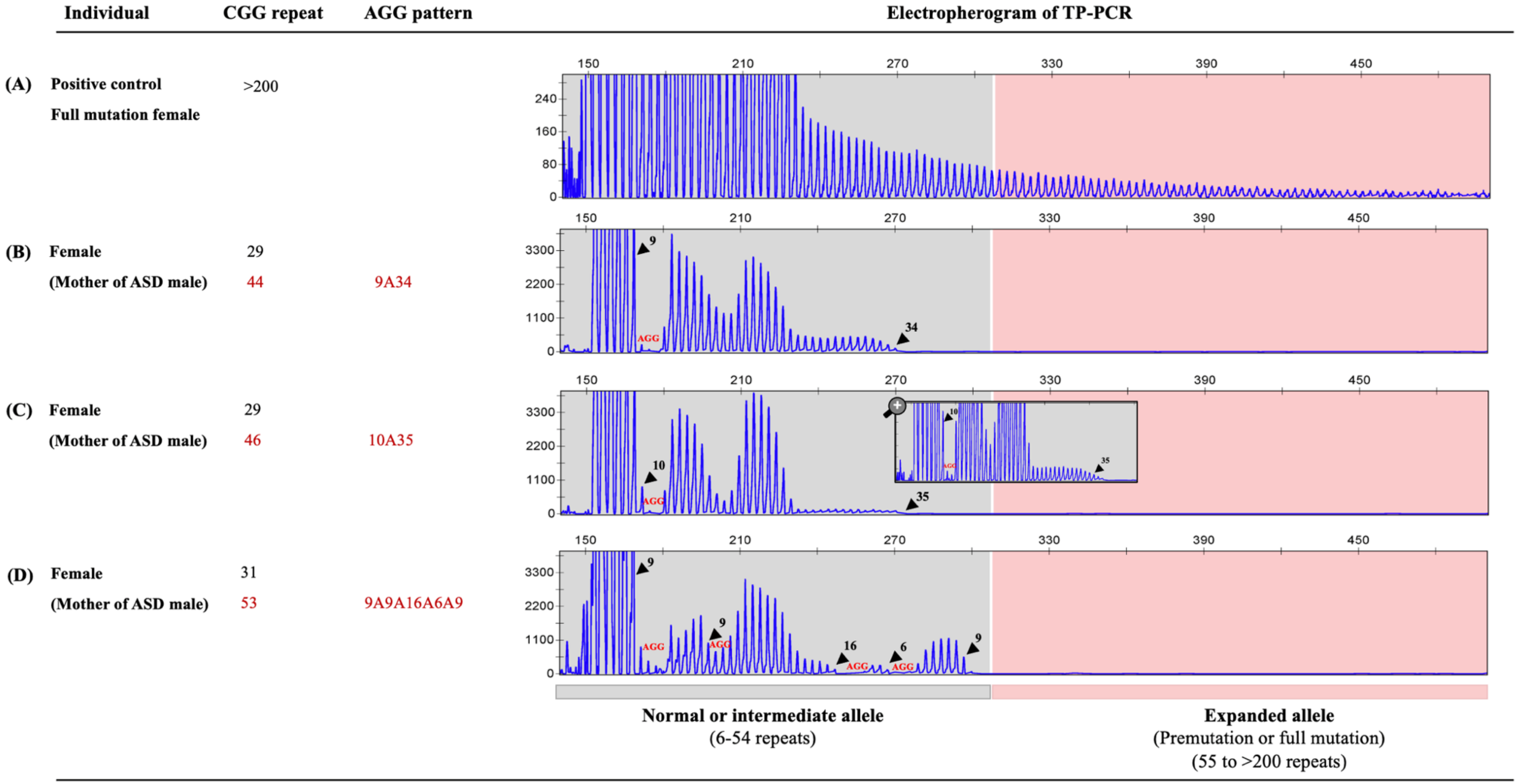

Supplementary Figure 3. Electropherogram of TP-PCR for *FMR1* CGG expanded alleles and AGG interruptions. Mothers of male patients carrying high normal or intermediate CGG alleles had at least one AGG interruption (A) full mutation female (positive control) showing serial peak (B) female with 29 and 44 CGG repeats: 44 repeats allele having one AGG interruption (C) female with 29 and 46 CGG repeats: 46 repeats allele having one AGG interruption. Superimposed close-up electropherogram shows the end of continuous stutter peaks of 46 repeats allele. (D) female with 31 and 53 CGG repeats: 53 repeats allele having four AGG interruptions. CGG repeats are based on the fluorescent PCR results. In heterozygous females, AGG interruption patterns can only be determined in larger repeat alleles because it is difficult to correctly identify AGG patterns in lower repeats alleles.
